# 3-Mesityl-2-oxo-1-oxaspiro­[4.4]non-3-en-4-yl 2-(4-chloro­phen­yl)-3-methyl­butyrate

**DOI:** 10.1107/S1600536808043043

**Published:** 2008-12-20

**Authors:** Chuan-Ming Yu, Yong Zhou, Jing-Li Cheng, Jin-Hao Zhao

**Affiliations:** a College of Chemical Engineering and Materials Science, Zhejiang University of Technology, Hangzhou 310032, People’s Republic of China; b College of Agriculture and Biotechnology, Zhejiang University, Hangzhou 310029, People’s Republic of China

## Abstract

In the title compound, C_28_H_31_ClO_4_, the five-membered cyclo­pentyl ring displays an envelope conformation with the atom at the flap position 0.519 (3) Å out of the mean plane formed by the other four atoms. The furan ring makes dihedral angles of 72.9 (1) and 82.4 (1)°, respectively, with the trimethyl- and chloro­phenyl rings. The dihedral angle between the two benzene rings is 15.3 (1)°. In the crystal, mol­ecules are linked through inter­molecular C—H⋯Cl hydrogen bonds, forming a chain running along the *b* axis.

## Related literature

For related compounds, see: Holmstead *et al.* (1978 ▶); Bayer Aktiengesellschaft (1995 ▶).
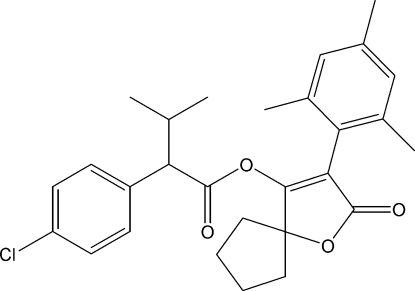


## Experimental

### 

#### Crystal data

C_28_H_31_ClO_4_
*M* *_r_* = 467.00
Monoclinic, 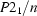

*a* = 13.9224 (11) Å
*b* = 14.2735 (12) Å
*c* = 14.3209 (11) Å
β = 113.9567 (17)°
*V* = 2600.7 (4) Å^3^
*Z* = 4
Mo *K*α radiation
μ = 0.18 mm^−1^
*T* = 296 (1) K
0.40 × 0.37 × 0.27 mm

#### Data collection

Rigaku R-AXIS RAPID diffractometer
Absorption correction: multi-scan (**ABSCOR**; Higashi, 1995 ▶) *T*_min_ = 0.927, *T*_max_ = 0.953
25138 measured reflections
5906 independent reflections
2647 reflections with *F* ^2^ > 2σ(*F* ^2^)
*R*_int_ = 0.054

#### Refinement

*R*[*F* ^2^ > 2σ(*F* ^2^)] = 0.046
*wR*(*F* ^2^) = 0.101
*S* = 1.00
5906 reflections
299 parameters
H-atom parameters constrained
Δρ_max_ = 0.54 e Å^−3^
Δρ_min_ = −0.54 e Å^−3^

### 

Data collection: *PROCESS-AUTO* (Rigaku, 1998 ▶); cell refinement: *PROCESS-AUTO*; data reduction: *CrystalStructure* (Rigaku/MSC, 2002 ▶) and Larson (1970 ▶); program(s) used to solve structure: *SIR97* (Altomare *et al.*, 1999 ▶); program(s) used to refine structure: *CRYSTALS* (Betteridge *et al.*, 2003 ▶); molecular graphics: *ORTEP-3* (Farrugia, 1997 ▶); software used to prepare material for publication: *CrystalStructure*.

## Supplementary Material

Crystal structure: contains datablocks General, I. DOI: 10.1107/S1600536808043043/is2366sup1.cif

Structure factors: contains datablocks I. DOI: 10.1107/S1600536808043043/is2366Isup2.hkl

Additional supplementary materials:  crystallographic information; 3D view; checkCIF report

## Figures and Tables

**Table 1:** Hydrogen-bond geometry (Å, °)

*D*—H⋯*A*	*D*—H	H⋯*A*	*D*⋯*A*	*D*—H⋯*A*
C17—H173⋯Cl1^i^	0.96	2.80	3.624 (5)	144

Symmetry code: (i) 
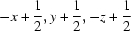
.

**Fig. 1.**
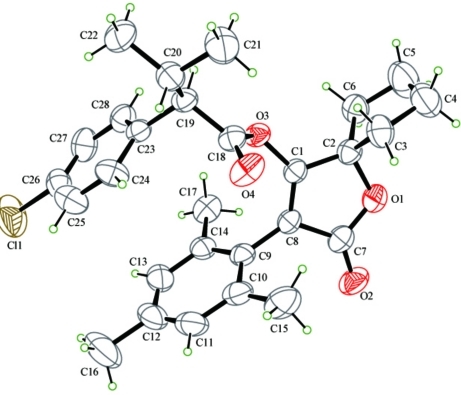
The molecular structure of (I), showing the atom-labelling scheme. Displacement ellipsoids are drawn at the 30% probability level.

**Fig. 2.**
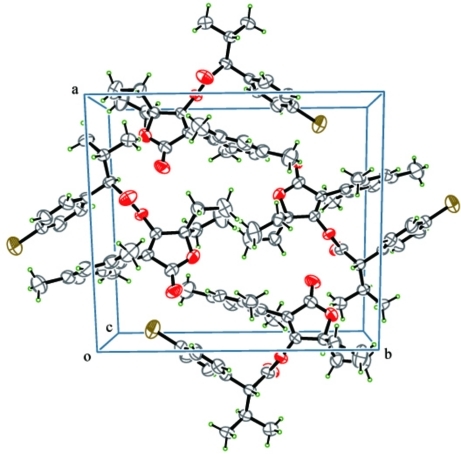
A packing diagram of the title compound, viewed along the c axis.

## supplementary crystallographic information

### Comment

2-(4-Chlorophenyl)-3-methylbutanoyl chloride is an intermediate in the synthesis of fenvalerate, an excellent insecticide (Holmstead *et al.*, 1978). 4-Hydroxyl-3-(2,4,6-trimethylphenyl)-1-oxaspiro[4,4]non-3-en-2-one is the key intermediate in preparing highly efficient acaricide-Spiromesifen developed by Bayer company (BAYER Aktiengesellschaft, 1995). As part of our continuing interest in the new acaricide design and synthesis, We have isolated the product, (I), of the condensation reaction of 2-(4-chlorophenyl)-3-methylbutanoyl chloride and 4-hydroxyl-3-(2,4,6-trimethylphenyl)-1-oxaspiro[4,4]non-3-en-2-one as colorless crystals suitable for X-ray analysis.

The molecular structure of (I) is shown in Fig. 1. The molecule contains two six-membered rings and two five-membered rings. The dihedral angles between the (C9—C14) and (C23—C28) rings, the (C9—C14) and furan rings, and the (C23—C28) and furan rings, are 15.3 (1), 72.9 (1) and 82.4 (1)°, respectively. The cyclopentyl ring displays an envelope conformation with C3 atom at the flap position 0.519 (3) Å out of the mean plane formed by the other four atoms. The title molecules are linked through intermolecular hydrogen bond of C17—H173···Cl1, forming chains running along the *b* axis. As expected, C1—C8, C7—O2 and C18—O4 are typically double bonds with bond distances of 1.327 (2), 1.207 (2) and 1.189 (3) Å. The bond distance of C7—C8 is 1.480 (2) Å, suggesting that carbonyl group on C7 has formed conjugate system with double bond on C8 and C1.

### Experimental

4-Hydroxyl-3-(2,4,6-trimethylphenyl)-1-oxaspiro[4,4]non-3-en-2-one (2.72 g, 10 mmol), 4-dimethylaminopyridine (0.58 g), triethylamine (1.31 g) and chloroform (100 ml) were added to a 250 ml round flask. Then the mixture was stirred and cooled to 273–278 K. Within 30 min 2-(4-chlorophenyl)-3-methylbutanoyl chloride (3.47 g) was added dropwise to the solution. The mixture was stirred at room temperature for 3 h and then 1% aqueous HCl was added. The organic layer was washed to neutral with water and dried over Na_2_SO_4_. After filtered and concentrated, the organic residue was purified by silica gel column chromatography, eluted with ethyl acetate–petroleum ether (1:30, *v*/*v*) to give a white solid (yield 79%, 3.69 g), which was then recrystallized from ethyl acetate/ethanol (1:1, *v*/*v*) to give colourless blocks.

### Refinement

The H atoms were geometrically placed (C—H = 0.93–0.98 Å) and refined as riding with *U*_iso_(H) = 1.2U_eq_(C) or 1.5U_eq_(methyl C). The methyl group was allowed to rotate, but not to tip, to best fit the electron density.

### Crystal data

**Table:**

C_28_H_31_ClO_4_	*F*(000) = 992.00
*M**_r_* = 467.00	*D*_x_ = 1.193 Mg m^−^^3^
Monoclinic, *P*2_1_/*n*	Mo *K*α radiation, λ = 0.71075 Å
Hall symbol: -P 2yn	Cell parameters from 11287 reflections
*a* = 13.9224 (11) Å	θ = 3.0–27.4°
*b* = 14.2735 (12) Å	µ = 0.18 mm^−^^1^
*c* = 14.3209 (11) Å	*T* = 296 K
β = 113.9567 (17)°	Chunk, colorless
*V* = 2600.7 (4) Å^3^	0.40 × 0.37 × 0.27 mm
*Z* = 4	

### Data collection

**Table:**

Rigaku R-AXIS RAPID diffractometer	2647 reflections with *F*^2^ > 2σ(*F*^2^)
Detector resolution: 10.00 pixels mm^-1^	*R*_int_ = 0.054
ω scans	θ_max_ = 27.4°
Absorption correction: multi-scan (*ABSCOR*; Higashi, 1995)	*h* = −18→15
*T*_min_ = 0.927, *T*_max_ = 0.953	*k* = −18→18
25138 measured reflections	*l* = −18→18
5906 independent reflections	

### Refinement

**Table:**

Refinement on *F*^2^	H-atom parameters constrained
*R*[*F*^2^ > 2σ(*F*^2^)] = 0.046	*w* = 1/[0.0001*F*_o_^2^ + 1.12σ(*F*_o_^2^)]/(4*F*_o_^2^)
*wR*(*F*^2^) = 0.101	(Δ/σ)_max_ < 0.001
*S* = 1.00	Δρ_max_ = 0.54 e Å^−^^3^
5906 reflections	Δρ_min_ = −0.54 e Å^−^^3^
299 parameters	Extinction correction: Larson (1970)
0 restraints	Extinction coefficient: 275 (22)

### Special details

**Table:**

Geometry. ENTER SPECIAL DETAILS OF THE MOLECULAR GEOMETRY
Refinement. Refinement using all reflections. The weighted *R*-factor (*wR*) and goodness of fit (*S*) are based on *F*^2^. *R*-factor (gt) are based on *F*. The threshold expression of *F*^2^ > 2.0 σ(*F*^2^) is used only for calculating *R*-factor (gt).

### Fractional atomic coordinates and isotropic or equivalent isotropic displacement parameters (Å^2^)

**Table:**

	*x*	*y*	*z*	*U*_iso_*/*U*_eq_	
Cl1	0.42195 (6)	−0.31031 (6)	0.32057 (8)	0.1664 (4)	
O1	0.37536 (12)	0.34277 (11)	0.19058 (12)	0.0873 (5)	
O2	0.24190 (12)	0.27677 (12)	0.05869 (11)	0.1041 (6)	
O3	0.54613 (10)	0.15522 (10)	0.32535 (9)	0.0693 (4)	
O4	0.61227 (11)	0.11705 (12)	0.21110 (12)	0.1006 (6)	
C1	0.46853 (17)	0.20939 (16)	0.25544 (16)	0.0639 (7)	
C2	0.47353 (17)	0.31237 (17)	0.27165 (16)	0.0718 (8)	
C3	0.56481 (19)	0.36173 (18)	0.25885 (19)	0.0962 (10)	
C4	0.5705 (2)	0.4562 (2)	0.3081 (2)	0.1294 (13)	
C5	0.5354 (2)	0.4401 (2)	0.3925 (2)	0.1513 (15)	
C6	0.48043 (18)	0.34765 (18)	0.37461 (17)	0.0897 (9)	
C7	0.3242 (2)	0.26762 (19)	0.13206 (19)	0.0806 (9)	
C8	0.38420 (16)	0.18106 (16)	0.17554 (14)	0.0657 (7)	
C9	0.35376 (16)	0.08583 (18)	0.13376 (17)	0.0695 (8)	
C10	0.36238 (18)	0.0607 (2)	0.04329 (18)	0.0876 (9)	
C11	0.3357 (2)	−0.0294 (2)	0.0068 (2)	0.1081 (11)	
C12	0.3023 (2)	−0.0946 (2)	0.0562 (2)	0.1111 (12)	
C13	0.29461 (17)	−0.0683 (2)	0.1455 (2)	0.0964 (10)	
C14	0.31929 (16)	0.0202 (2)	0.18571 (18)	0.0738 (8)	
C15	0.4000 (2)	0.1298 (2)	−0.01408 (17)	0.1207 (11)	
C16	0.2754 (2)	−0.1933 (2)	0.0138 (2)	0.1759 (15)	
C17	0.31119 (16)	0.04449 (17)	0.28478 (16)	0.0943 (9)	
C18	0.61283 (16)	0.10648 (16)	0.29364 (18)	0.0713 (8)	
C19	0.67873 (16)	0.04038 (16)	0.37635 (14)	0.0688 (7)	
C20	0.78659 (17)	0.02336 (17)	0.37580 (17)	0.0836 (8)	
C21	0.84596 (18)	0.11507 (19)	0.3864 (2)	0.1194 (11)	
C22	0.84973 (17)	−0.04342 (17)	0.46174 (19)	0.1202 (10)	
C23	0.61429 (14)	−0.04848 (16)	0.36195 (16)	0.0640 (7)	
C24	0.59659 (18)	−0.1081 (2)	0.28109 (17)	0.0914 (9)	
C25	0.5382 (2)	−0.1883 (2)	0.2682 (2)	0.1062 (11)	
C26	0.49659 (18)	−0.20939 (19)	0.3369 (2)	0.0921 (10)	
C27	0.5106 (2)	−0.1518 (2)	0.4159 (2)	0.1004 (11)	
C28	0.57019 (19)	−0.07207 (19)	0.42889 (16)	0.0817 (9)	
H11	0.3408	−0.0462	−0.0538	0.130*	
H13	0.2718	−0.1123	0.1799	0.116*	
H19	0.6889	0.0679	0.4424	0.083*	
H20	0.7769	−0.0056	0.3105	0.100*	
H24	0.6248	−0.0937	0.2341	0.110*	
H25	0.5272	−0.2278	0.2132	0.127*	
H27	0.4802	−0.1658	0.4613	0.120*	
H28	0.5809	−0.0332	0.4843	0.098*	
H31	0.6298	0.3273	0.2932	0.115*	
H32	0.5508	0.3686	0.1871	0.115*	
H41	0.6420	0.4799	0.3352	0.155*	
H42	0.5244	0.5005	0.2589	0.155*	
H51	0.5958	0.4394	0.4577	0.182*	
H52	0.4877	0.4896	0.3926	0.182*	
H61	0.5201	0.3039	0.4283	0.108*	
H62	0.4105	0.3552	0.3730	0.108*	
H151	0.4463	0.1743	0.0330	0.145*	
H152	0.3408	0.1620	−0.0638	0.145*	
H153	0.4369	0.0970	−0.0480	0.145*	
H161	0.3352	−0.2204	0.0067	0.211*	
H162	0.2172	−0.1911	−0.0517	0.211*	
H163	0.2569	−0.2307	0.0598	0.211*	
H171	0.2736	−0.0041	0.3020	0.113*	
H172	0.3804	0.0502	0.3381	0.113*	
H173	0.2744	0.1028	0.2772	0.113*	
H211	0.9155	0.1022	0.3911	0.143*	
H212	0.8505	0.1471	0.4469	0.143*	
H213	0.8094	0.1537	0.3277	0.143*	
H221	0.8144	−0.1028	0.4510	0.144*	
H222	0.8562	−0.0177	0.5259	0.144*	
H223	0.9184	−0.0519	0.4624	0.144*	

### Atomic displacement parameters (Å^2^)

**Table:**

	*U*^11^	*U*^22^	*U*^33^	*U*^12^	*U*^13^	*U*^23^
Cl1	0.1490 (7)	0.0969 (6)	0.2528 (10)	−0.0309 (5)	0.0811 (7)	−0.0047 (6)
O1	0.0850 (11)	0.0733 (12)	0.0947 (10)	0.0176 (10)	0.0273 (9)	0.0109 (9)
O2	0.0821 (10)	0.1143 (15)	0.0921 (11)	0.0278 (10)	0.0108 (9)	0.0219 (9)
O3	0.0591 (8)	0.0761 (11)	0.0651 (8)	0.0144 (8)	0.0172 (7)	−0.0001 (7)
O4	0.0949 (11)	0.1351 (16)	0.0831 (10)	0.0363 (10)	0.0478 (9)	0.0247 (10)
C1	0.0606 (14)	0.0651 (17)	0.0666 (13)	0.0096 (13)	0.0264 (11)	0.0041 (12)
C2	0.0654 (15)	0.0737 (19)	0.0756 (15)	0.0058 (13)	0.0279 (12)	0.0049 (13)
C3	0.0989 (19)	0.083 (2)	0.1177 (19)	−0.0086 (16)	0.0549 (16)	0.0063 (16)
C4	0.158 (2)	0.103 (2)	0.130 (2)	−0.038 (2)	0.060 (2)	−0.015 (2)
C5	0.239 (3)	0.108 (2)	0.123 (2)	−0.038 (2)	0.089 (2)	−0.021 (2)
C6	0.0990 (18)	0.083 (2)	0.0945 (17)	0.0096 (16)	0.0468 (14)	−0.0046 (14)
C7	0.0748 (17)	0.085 (2)	0.0815 (16)	0.0142 (17)	0.0315 (13)	0.0117 (16)
C8	0.0597 (14)	0.0692 (18)	0.0674 (14)	0.0103 (13)	0.0250 (11)	0.0072 (13)
C9	0.0558 (13)	0.0770 (19)	0.0643 (15)	0.0092 (13)	0.0128 (11)	−0.0016 (14)
C10	0.0793 (16)	0.106 (2)	0.0632 (16)	0.0215 (16)	0.0138 (12)	−0.0028 (17)
C11	0.101 (2)	0.120 (2)	0.0779 (19)	0.018 (2)	0.0097 (15)	−0.030 (2)
C12	0.0754 (18)	0.095 (2)	0.122 (2)	0.0022 (18)	−0.0015 (18)	−0.035 (2)
C13	0.0639 (15)	0.084 (2)	0.118 (2)	−0.0059 (15)	0.0132 (15)	−0.0046 (18)
C14	0.0520 (13)	0.082 (2)	0.0751 (15)	0.0024 (13)	0.0134 (11)	−0.0015 (15)
C15	0.144 (2)	0.149 (2)	0.0794 (16)	0.029 (2)	0.0556 (17)	0.0108 (18)
C16	0.145 (2)	0.126 (2)	0.212 (3)	−0.025 (2)	0.026 (2)	−0.079 (2)
C17	0.0816 (16)	0.109 (2)	0.0970 (17)	0.0076 (15)	0.0410 (13)	0.0154 (15)
C18	0.0582 (14)	0.0827 (18)	0.0715 (15)	0.0098 (13)	0.0250 (12)	0.0012 (14)
C19	0.0604 (13)	0.0752 (17)	0.0644 (12)	0.0112 (13)	0.0186 (10)	−0.0014 (11)
C20	0.0618 (14)	0.0789 (18)	0.1046 (17)	0.0063 (14)	0.0282 (13)	−0.0084 (14)
C21	0.0784 (17)	0.106 (2)	0.169 (2)	−0.0032 (17)	0.0455 (17)	−0.0001 (19)
C22	0.0646 (15)	0.108 (2)	0.152 (2)	0.0190 (16)	0.0073 (15)	0.0158 (19)
C23	0.0587 (13)	0.0717 (17)	0.0555 (12)	0.0099 (12)	0.0169 (11)	0.0017 (13)
C24	0.0884 (18)	0.111 (2)	0.0807 (17)	−0.0201 (17)	0.0403 (13)	−0.0232 (16)
C25	0.097 (2)	0.111 (2)	0.106 (2)	−0.0161 (18)	0.0368 (17)	−0.0342 (18)
C26	0.0726 (16)	0.072 (2)	0.125 (2)	−0.0007 (14)	0.0333 (16)	0.0003 (18)
C27	0.114 (2)	0.087 (2)	0.119 (2)	0.0104 (19)	0.0669 (17)	0.0220 (19)
C28	0.0943 (18)	0.081 (2)	0.0755 (15)	0.0144 (16)	0.0407 (14)	0.0085 (14)

### Geometric parameters (Å, °)

**Table:**

Cl1—C26	1.736 (2)	C26—C27	1.347 (4)
O1—C2	1.455 (2)	C27—C28	1.376 (4)
O1—C7	1.370 (2)	C3—H31	0.970
O2—C7	1.207 (2)	C3—H32	0.970
O3—C1	1.374 (2)	C4—H41	0.970
O3—C18	1.377 (3)	C4—H42	0.970
O4—C18	1.189 (3)	C5—H51	0.970
C1—C2	1.485 (3)	C5—H52	0.970
C1—C8	1.327 (2)	C6—H61	0.970
C2—C3	1.528 (3)	C6—H62	0.970
C2—C6	1.524 (3)	C11—H11	0.930
C3—C4	1.509 (3)	C13—H13	0.930
C4—C5	1.495 (5)	C15—H151	0.960
C5—C6	1.495 (4)	C15—H152	0.960
C7—C8	1.480 (3)	C15—H153	0.960
C8—C9	1.476 (3)	C16—H161	0.960
C9—C10	1.396 (3)	C16—H162	0.960
C9—C14	1.397 (3)	C16—H163	0.960
C10—C11	1.382 (4)	C17—H171	0.960
C10—C15	1.507 (4)	C17—H172	0.960
C11—C12	1.360 (5)	C17—H173	0.960
C12—C13	1.377 (5)	C19—H19	0.980
C12—C16	1.519 (4)	C20—H20	0.980
C13—C14	1.374 (4)	C21—H211	0.960
C14—C17	1.508 (3)	C21—H212	0.960
C18—C19	1.501 (2)	C21—H213	0.960
C19—C20	1.524 (3)	C22—H221	0.960
C19—C23	1.519 (3)	C22—H222	0.960
C20—C21	1.523 (3)	C22—H223	0.960
C20—C22	1.522 (3)	C24—H24	0.930
C23—C24	1.376 (3)	C25—H25	0.930
C23—C28	1.374 (3)	C27—H27	0.930
C24—C25	1.372 (4)	C28—H28	0.930
C25—C26	1.364 (5)		
			
C2—O1—C7	109.97 (17)	C5—C4—H41	110.4
C1—O3—C18	118.69 (17)	C5—C4—H42	110.4
O3—C1—C2	118.02 (15)	H41—C4—H42	109.5
O3—C1—C8	128.0 (2)	C4—C5—H51	109.8
C2—C1—C8	113.91 (18)	C4—C5—H52	109.8
O1—C2—C1	101.61 (15)	C6—C5—H51	109.8
O1—C2—C3	108.93 (18)	C6—C5—H52	109.8
O1—C2—C6	110.08 (19)	H51—C5—H52	109.5
C1—C2—C3	115.2 (2)	C2—C6—H61	110.2
C1—C2—C6	117.3 (2)	C2—C6—H62	110.2
C3—C2—C6	103.59 (18)	C5—C6—H61	110.2
C2—C3—C4	104.2 (2)	C5—C6—H62	110.2
C3—C4—C5	105.6 (2)	H61—C6—H62	109.5
C4—C5—C6	108.0 (2)	C10—C11—H11	118.8
C2—C6—C5	106.5 (2)	C12—C11—H11	118.8
O1—C7—O2	121.5 (2)	C12—C13—H13	118.7
O1—C7—C8	109.38 (16)	C14—C13—H13	118.7
O2—C7—C8	129.1 (2)	C10—C15—H151	109.5
C1—C8—C7	105.02 (19)	C10—C15—H152	109.5
C1—C8—C9	129.65 (19)	C10—C15—H153	109.5
C7—C8—C9	125.32 (16)	H151—C15—H152	109.5
C8—C9—C10	119.6 (2)	H151—C15—H153	109.5
C8—C9—C14	120.4 (2)	H152—C15—H153	109.5
C10—C9—C14	120.0 (2)	C12—C16—H161	109.5
C9—C10—C11	118.6 (2)	C12—C16—H162	109.5
C9—C10—C15	121.2 (2)	C12—C16—H163	109.5
C11—C10—C15	120.2 (2)	H161—C16—H162	109.5
C10—C11—C12	122.4 (3)	H161—C16—H163	109.5
C11—C12—C13	118.0 (3)	H162—C16—H163	109.5
C11—C12—C16	120.5 (3)	C14—C17—H171	109.5
C13—C12—C16	121.6 (3)	C14—C17—H172	109.5
C12—C13—C14	122.6 (3)	C14—C17—H173	109.5
C9—C14—C13	118.4 (2)	H171—C17—H172	109.5
C9—C14—C17	121.3 (2)	H171—C17—H173	109.5
C13—C14—C17	120.3 (2)	H172—C17—H173	109.5
O3—C18—O4	122.08 (18)	C18—C19—H19	108.2
O3—C18—C19	109.6 (2)	C20—C19—H19	108.2
O4—C18—C19	128.3 (2)	C23—C19—H19	108.2
C18—C19—C20	112.6 (2)	C19—C20—H20	108.5
C18—C19—C23	106.25 (15)	C21—C20—H20	108.5
C20—C19—C23	113.27 (19)	C22—C20—H20	108.5
C19—C20—C21	111.0 (2)	C20—C21—H211	109.5
C19—C20—C22	110.0 (2)	C20—C21—H212	109.5
C21—C20—C22	110.24 (16)	C20—C21—H213	109.5
C19—C23—C24	121.7 (2)	H211—C21—H212	109.5
C19—C23—C28	121.1 (2)	H211—C21—H213	109.5
C24—C23—C28	117.2 (2)	H212—C21—H213	109.5
C23—C24—C25	121.5 (2)	C20—C22—H221	109.5
C24—C25—C26	119.3 (2)	C20—C22—H222	109.5
Cl1—C26—C25	119.5 (2)	C20—C22—H223	109.5
Cl1—C26—C27	119.6 (2)	H221—C22—H222	109.5
C25—C26—C27	120.8 (2)	H221—C22—H223	109.5
C26—C27—C28	119.5 (3)	H222—C22—H223	109.5
C23—C28—C27	121.6 (2)	C23—C24—H24	119.2
C2—C3—H31	110.8	C25—C24—H24	119.2
C2—C3—H32	110.8	C24—C25—H25	120.4
C4—C3—H31	110.8	C26—C25—H25	120.4
C4—C3—H32	110.8	C26—C27—H27	120.3
H31—C3—H32	109.5	C28—C27—H27	120.3
C3—C4—H41	110.4	C23—C28—H28	119.2
C3—C4—H42	110.4	C27—C28—H28	119.2
			
C2—O1—C7—O2	−178.3 (2)	C8—C9—C10—C15	−1.4 (3)
C2—O1—C7—C8	3.1 (3)	C8—C9—C14—C13	−178.19 (18)
C7—O1—C2—C1	−3.2 (2)	C8—C9—C14—C17	0.5 (2)
C7—O1—C2—C3	118.8 (2)	C10—C9—C14—C13	0.0 (2)
C7—O1—C2—C6	−128.2 (2)	C10—C9—C14—C17	178.74 (18)
C1—O3—C18—O4	7.7 (2)	C14—C9—C10—C11	0.3 (3)
C1—O3—C18—C19	−170.62 (17)	C14—C9—C10—C15	−179.6 (2)
C18—O3—C1—C2	−115.3 (2)	C9—C10—C11—C12	−0.6 (3)
C18—O3—C1—C8	68.4 (3)	C15—C10—C11—C12	179.4 (2)
O3—C1—C2—O1	−174.4 (2)	C10—C11—C12—C13	0.4 (4)
O3—C1—C2—C3	68.0 (2)	C10—C11—C12—C16	−179.1 (2)
O3—C1—C2—C6	−54.4 (3)	C11—C12—C13—C14	−0.0 (3)
O3—C1—C8—C7	175.8 (2)	C16—C12—C13—C14	179.5 (2)
O3—C1—C8—C9	−5.6 (4)	C12—C13—C14—C9	−0.2 (3)
C2—C1—C8—C7	−0.6 (3)	C12—C13—C14—C17	−178.9 (2)
C2—C1—C8—C9	177.9 (2)	O3—C18—C19—C20	−149.74 (17)
C8—C1—C2—O1	2.4 (3)	O3—C18—C19—C23	85.7 (2)
C8—C1—C2—C3	−115.2 (2)	O4—C18—C19—C20	32.0 (3)
C8—C1—C2—C6	122.4 (2)	O4—C18—C19—C23	−92.5 (2)
O1—C2—C3—C4	82.6 (2)	C18—C19—C20—C21	57.2 (2)
O1—C2—C6—C5	−91.6 (2)	C18—C19—C20—C22	179.54 (18)
C1—C2—C3—C4	−164.07 (18)	C18—C19—C23—C24	71.0 (2)
C1—C2—C6—C5	152.9 (2)	C18—C19—C23—C28	−108.2 (2)
C3—C2—C6—C5	24.7 (2)	C20—C19—C23—C24	−53.1 (2)
C6—C2—C3—C4	−34.6 (2)	C20—C19—C23—C28	127.7 (2)
C2—C3—C4—C5	31.7 (2)	C23—C19—C20—C21	177.81 (18)
C3—C4—C5—C6	−16.4 (2)	C23—C19—C20—C22	−59.9 (2)
C4—C5—C6—C2	−5.4 (2)	C19—C23—C24—C25	−179.93 (19)
O1—C7—C8—C1	−1.5 (3)	C19—C23—C28—C27	179.2 (2)
O1—C7—C8—C9	179.8 (2)	C24—C23—C28—C27	−0.1 (2)
O2—C7—C8—C1	180.0 (2)	C28—C23—C24—C25	−0.6 (3)
O2—C7—C8—C9	1.3 (5)	C23—C24—C25—C26	0.1 (3)
C1—C8—C9—C10	−106.0 (3)	C24—C25—C26—Cl1	179.56 (19)
C1—C8—C9—C14	72.2 (3)	C24—C25—C26—C27	1.1 (3)
C7—C8—C9—C10	72.4 (3)	Cl1—C26—C27—C28	179.72 (19)
C7—C8—C9—C14	−109.4 (2)	C25—C26—C27—C28	−1.9 (3)
C8—C9—C10—C11	178.6 (2)	C26—C27—C28—C23	1.3 (3)

### Hydrogen-bond geometry (Å, °)

**Table:**

*D*—H···*A*	*D*—H	H···*A*	*D*···*A*	*D*—H···*A*
C17—H173···Cl1^i^	0.96	2.80	3.624 (5)	144

Symmetry codes: (i) −*x*+1/2, *y*+1/2, −*z*+1/2.
